# The Not-So-Strange Case of Dr. Jekyll and Mr. Hyde in Antibiotic Research: An Interdisciplinary Opportunity

**DOI:** 10.3390/antibiotics10010019

**Published:** 2020-12-28

**Authors:** Lorenzo Servitje

**Affiliations:** Department of English, Health, Medicine, and Society Program, Lehigh University, Bethlehem, PA 18015, USA; los317@lehigh.edu; Tel.: +1-610-758-3322

**Keywords:** virulence, pathogenesis, antibiotic resistance, anti-virulence, interdisciplinarity, *C. diphtheriae*

## Abstract

Literary-rhetorical devices like figurative language and analogy can help explain concepts that exceed our capacity to grasp intuitively. It is not surprising these devices are used to discuss virulence, pathogenesis, and antibiotics. Allusions to Robert Louis Stevenson’s *Strange Case of Dr. Jekyll and Mr. Hyde* seem to be used with particular frequency in research pertaining to pathogens, especially in studies contemporary with our evolving understanding of antibiotic resistance. More recent references to the text have appeared in research parsing definitions of virulence and acknowledging the role of anti-virulence in future therapeutics. While it is obvious that scientists invoke Stevenson’s story for stylistic purposes, its use could go beyond the stylistic—and might even generate rhetorical and imaginative possibilities for framing research. This perspective discusses the first published allusion to *Jekyll and Hyde* in reference to virulence and pathogenesis; comments on a select number of specific instances of *Jekyll and Hyde* in contemporary scientific literature; briefly contextualizes the novel; and concludes with the implications of a more productive engagement with humanistic disciplines in the face of antibiotic resistance.

## 1. Introduction

Virulence is a notoriously expansive, mutable term in its uses across different disciplines and over time [1,2,3,4,5,6,7,8,9,10]. Casadevall and Pirofski suggest that it “encompasses everything that contributes to making microbes pathogenic” (p. 2) [1]. Virulence, however, has a non-constant quality to it. It is an unusual microbial property because it does not “define an independent determinant of microbial activity, or characteristics” (p. 2) [1]. And while it is subject to qualitative and quantitative measures, those measures themselves are variable, contingent upon microbe, host, environmental, and social factors—and the entangled interactions amongst them [1,2,3,4]. Pathogens can reside in hosts and their microbiome without signs or symptoms, and commensals can harbor both genes that express virulence factors and genes that code for antibiotic resistance mechanisms [11]. Terms like virulence and pathogen define what and how we categorize and respond to microbes in clinical practice and biomedical research. Thus, their definitions have, do, and will impact the use of antibiotics and antibiotic-alternative therapies like anti-virulence in the future. We should attend to and expand our consideration of how these terms are thought of and written about. Figurative language and analogy are literary-rhetorical devices that can help explain concepts that exceed our capacity to grasp intuitively. It is not surprising they are used to discuss virulence, pathogenesis, and antibiotics.

A notable example of this is the recurrent use of “Jekyll and Hyde” in research pertaining to pathogens, and especially in research focusing on the accretional evolution of antibiotic resistance. Consider, for instance, the July 2019 special issue of the *Journal of Molecular Biology*, “Jekyll and Hyde: Bugs with Double Personalities that Muddle the Distinction between Commensal and Pathogen” [12], whose guest editors invite readers to expand their understanding of their objects of inquiry: “We hope that the articles presented in the special issue will inspire you to delve deeper into the heterogeneity and dualistic Jekyll-and-Hyde potential of the microbes that you study” (p. 2912) [12].

Deployments of *Jekyll and Hyde* occur in the context of research that underscores the gray zone where virulence is a mutable and relational property of pathogens, determined by a number of variables in a microbe, its hosts, and their environments [4,12]. However, in those deployments it is often, but not always, the case that the “Jekyll and Hyde” allusion itself stands in for simplistic dualism—good vs. bad, pathogen or commensal (or saprophytic)—and carries with it a somewhat reductive, albeit popular, reading of Stevenson’s novel: the Janus-faced nature of humankind. Yet since at least the late 1980s, with the publication of *Dr. Jekyll and Mr. Hyde After One Hundred Years*, most humanities-based readings of the novel, many stemming from nuanced theorizations of the Gothic genre and featuring extensive research into contemporaneous science and culture, document how *Jekyll and Hyde* in fact undercuts any attempt at discretizing the identity categories it seems to support on the surface: evolved and degenerate; good and evil; normal and pathological. This is not to say biomedical researchers are using literature “incorrectly”; rather, it is to say that literature could be doing much more work for and with those researchers than they might realize. The above not-so-strange case of “Jekyll and Hyde” and bacteria serves as one small example: how would using *Jekyll and Hyde* to think about mutability and contextuality instead of duality change the framing of their conclusions? The perspective that follows will discuss the first published allusion to *Jekyll and Hyde* in reference to virulence and pathogenesis; cite and comment on a select number of references to the novel in contemporary scientific literature; briefly contextualize the novel and its genre; and conclude with the implications of a more symbiotic and fruitful engagement among scientific and humanistic disciplines in the face of antibiotic resistance.

## 2. Jekyll and Hyde, or Civilization and the Microbe

In order to properly understand the significance of the *Jekyll and Hyde* allusions in antibiotic and microbiological research publications, it is important to contextualize their origin. This is critical for three reasons. First, the initial instance of this usage occurs right at the cusp of antimicrobial chemotherapy’s emergence. Second, it contains an extended conceit that is in line with our contemporary understanding of the dynamic nature of virulence with respect to environment and microbe. And third, it falls into the anthropocentric trap—in language and rhetoric—of subordinating microbes to human ends, a logic that has facilitated the overuse of antibiotics from the very moment of their inception.

The first time *Jekyll and Hyde* was deployed in published scientific prose can be found in the popular science book *Civilization and the Microbe* (1923), authored by Arthur Isaac Kendall, an influential American bacteriologist of the early twentieth century. Kendall’s book documented the knowledge of microbiology, immunology, and pathology—with a specific focus on bacteria—in straightforward language. Published a few years shy of Fleming’s discovery of penicillin (1928) and the introduction of sulfonamides (1932) to the pharmacopoeia, but two decades into the use of organoarsenicals such as axtoyl (1905) and Salvarsan (1910) [13,14], Kendall, among others, recognized that the relationship between humans and microbes entailed positive or ambivalent vectors of influence, and amounted to more than just the need for humans to eradicate pathogens. It is worth acknowledging, however, that the tenor of his writing still evidenced an anthropocentric view of that relationship (not uncommon for the period), where microbes would be eliminated or “tamed” for technological repurposing: “Civilization and the microbe go hand in hand, but the germ must be investigated, and the vast power locked up in the life-processes of these ever-toiling agents must be segregated and utilized to promote the prosperity and the happiness of the human race” (p. xvii) [15].

After discussing nutritive substances, Kendall has a subheading of a chapter entitled “The Microbic Jekyll and Hyde.” He writes: “The story of Dr. Jekyll and Mr. Hyde, that strange and imaginary conception of a dual human personality, has its actual realization, and far more striking and realistic, in this simple experiment upon the energy requirements of the diphtheria bacillus. In plain broth the microbe produces a potent toxin which confers on the bacillus its formidableness in producing disease. The simple addition of glucose as a readily utilizable source of energy for the organism so changes the nature of its growth products that they are not only no longer toxic—they are potentially possessed of food value. They are actually the chemical equivalent of buttermilk” (p. 59) [15]. Kendall’s discussion of *C. diphtheriae* is reflective of the fact that the bacterium does not grow well in acidic conditions; in an overly glucose-rich environment, it will produce more pyruvic acid and lactic acid (the latter along with glucose seeming to represent “buttermilk”), creating a negative feedback mechanism. Kendall is likely informed by Theobald Smith and other researchers from the late nineteenth and early twentieth centuries who experimented with different growing environments, along with the work of Fredrick Loeffler, who isolated apparently indistinguishable strains of *C. diphtheriae*, an avirulent strain from healthy individuals and a toxigenic (virulent) strain in patients exhibiting symptoms [16,17,18].

Notable here is that, though there is a clear binary, Jekyll being avirulent and Hyde being virulent, it is the mutable milieu (in vitro with a high glucose concentration) that changed the bacterium. This is not an instance of antimicrobial chemotherapy—at the time dominated by organoarsenicals—but one in which virulence is attenuated, an early example of anti-virulence thinking, I would suggest. It is important to consider the environment and the microbe in a causal relationship with respect to Jekyll and Hyde here. Kendall uses glucose to alter the Hyde-virulent diphtheriae into the Jekyll-avirulent diphtheriae—the monster is civilized, “promoting the prosperity and happiness of the human race” by producing the most innocuous of all substances: buttermilk. This is a reversal of the complex function of environment in the original novel, where the corruption, pollution, and matter out of place—literal and metaphorical—of late Victorian London give birth to Edward Hyde. Taylor, for instance, has contextualized the novel with respect to anthropogenic pollution and shown the constitutive relationship between humans and their technological effects on nature; in this view, humans produce the toxic environments of which Hyde is a consequential effect and embodiment of [19]. Hensley, likewise, considers the novel’s setting, and shows how the novel positions Hyde as the parasitic effect of the British empire’s material downturn [20]. In the novel, then, technological evolution (industrialization, urbanization, and colonization) catalyzes human devolution through environmental mediation (pollution and urban decay) [19,20,21]. As will be discussed in the final section, Gothic fiction like *Jekyll and Hyde* renders such dualisms meaningless in themselves. Consequently, we can read Kendall’s extended analogy ironically, as he uses *Jekyll and Hyde* to do that which the original novel critiques: “civilize” and repurpose nature (pathogens, in his case, or baser human instincts, in the case of Dr. Jekyll). His allusion says more about the zeitgeist of technological utopianism that would characterize the golden age of antibiotics at mid-century than it does about *C. diphtheriae*. Two decades after Kendall’s *Civilization and the Microbe*, following the mass production and distribution of penicillin and streptomycin, Boris Sokoloff expressed a similar sentiment in his widely read popular science book *The Miracle Drugs* (1949): “The goal is simply to live in a world without menacing microbes; to have all disease-producing microbes rendered harmless and domesticated… Will such a world exist? We believe so” (p. 254) [22]. Compare this goal to the ill-fated imperative of the fictional Victor Frankenstein to “banish disease from the human frame and render man invulnerable to any but a violent death” (p. 55) [23]—a project that ultimately threatens the human race—and the resonance with antibiotic resistance grows stronger. From our twenty-first-century vantage, it is clear that Kendall, Sokoloff, and Victor Frankenstein’s grand visions were not only short-sighted; they were catastrophic.

## 3. Novel Allusions in the Age of Resistance and Anti-Virulence

Since *Civilization and the Microbe* there have been at least thirty instances of “Jekyll and Hyde” in scientific literature discussing microbiology, immunology, pharmacology, and bioengineering, the majority in the past three decades, including one from an article in *Antibiotics* published in 2020 [24,25,26,27,28,29,30,31]. Apart from the previously cited instances, a sampling from across fields provides a useful survey.

A helpful example to begin with is Gray et al.’s “How the Bacterial Pathogen *Listeria monocytogenes* Mediates the Switch from Environmental Dr. Jekyll to Pathogenic Mr. Hyde.” The authors weave the novel’s protagonist and antagonist thematically throughout their subheadings, as they explain the “switching” function of transcriptional activator positive regulatory factor A (*prfa*) and the environmental factors that influence virulence gene expression in *L. monocytogenes*. They conclude by commenting that it “therefore appears that *L. monocytogenes* must maintain a balance between life in the outside environment and life within the host; thus, bacteria that can undergo the switch back to the humble Dr. Jekyll form may be favored over the evolution of increasingly dangerous Mr. Hydes” (p. 2509) [32]. This is a common deployment of the novel, where Jekyll denotes something like a “peaceful saprophyte” and Hyde refers to a “deadly pathogen.” Because *Jekyll and Hyde* challenges assumptions of progressive human evolution, especially the common assumption that change is teleological and we always evolve to more “civilized” and enlightened forms (aiming toward some perfect end), this article’s description of evolution favoring the mutable pathogen that can switch in response to environment inherently challenges the avirulence hypothesis, which posits that pathogens evolve towards mutual tolerance with their hosts. This was a popular theory beginning in the early twentieth century, one that gained credibility after the effort to eradicate the rabbit population in Australia in the 1950s provided seemingly natural experimental proof: the myxoma virus introduced to rabbits there initially had a nearly 100% mortality rate, but within a few years that initial strain became significantly less lethal and virulent [7]. The avirulence hypothesis has since been challenged by more complex trade-off models, although it has proved difficult to reconcile empirical and theoretical models [33].

Keen’s “Paradigms of Pathogenesis: Targeting the Mobile Genetic Elements of Disease” uses the allusion as a qualifier for a specific kind of pathogen: “Evolutionarily speaking, there seem to be at least two broad categories of pathogenic bacteria: obligate pathogens that have evolved over time to become irreversibly specialized parasites and ‘Jekyll-and-Hyde pathogens,’ still closely related to free-living bacteria, that have been rapidly but reversibly made pathogenic by mobile genetic elements” (p. 1) [34]. The author-defined denomination is repeated six more times and was later cited by Méthot and Alizon in the inaugural issue of *Virulence* [4]. Keen hypothesizes that mobile genetic elements, such as bacteriophages and plasmids, mediate pathogenicity in “Jekyll-and-Hyde” pathogens like *E. coli*, *C. diphtheriae*, and *V. cholerae*. He contends that this distinction “between full-scale genetic re-wiring and subtle genetic fine-tuning represents a fundamental contrast that may shed light on the past, present, and future evolution of pathogenic bacteria” (p. 1) [34]. Indeed, these comments mirror more nuanced readings of the novel, whose narrative consistently undermines Jekyll’s desire to externalize the evil doppelganger Hyde. Moreover, the “fundamental contrast” Keen insists on is not representing pathogenic and non-pathogenic bacteria, but rather the two sides of the heuristic we use to understand how they switch—rather than a full-scale change in essence (“genetic rewiring”), he posits an environmentally mediated (external actor) modulation. This comparison, I would suggest, maps closely onto the ways *Jekyll and Hyde* itself is used to understand “Jekyll-and-Hyde” pathogens: one way is a simple dualism that signifies “good” and “bad” bacteria (a use that is often less indicative of the modulations and relations authors describe in their research); the other is subtler, more self-reflexive, and draws attention to the way the novel reflects complexity, indeterminacy, and inextricable contextual relation.

Hallet’s “Playing Dr. Jekyll and Mr. Hyde: Combined Mechanisms of Phase Variation in Bacteria” begins with an epigraph “If he be Mr. Hyde, I shall be Mr. Seek!” a line from early in the novel. The line belongs to one of the central protagonists, Utterson, a close friend of Dr. Jekyll, as he vows to discover the meaning of strange rumors he hears about the doctor’s new acquaintance. The paper concludes by contending that future research on the ability of bacteria to vary protein expression in the face of rapidly evolving environments “will continue to help micropathologists to hold the promise made by Dr. Jekyll in the novel by RL Stevenson: ‘I will tell you one thing: the moment I choose, I can be rid of Mr. Hyde. I will give my hand upon that’” (p. 570) [35]. In this case, the titular reference places the microbiologist in the role of Dr. Jekyll, who completes an “examination of the genomes of pathogenic bacterial species [that] reveals the existence of multiple mechanisms that allow continuous evolution through the deletion, duplication and lateral acquisition of genetic material” (p. 570) [35]. Hyde denotes the ability of certain bacteria to produce reversible and high frequency mutations—the kind of bacteria Gray et al. model as being able to shift between both “personas”—but here the allusion operates as paronomasia, more colloquially known as a pun. The article presents a compelling symmetrical coherence with its bookended quotes from the novel; yet given the context of antibiotic resistance and its unwitting promotion by utopian thinking, ridding ourselves of Mr. Hyde whenever we choose might not be the most prudent statement to close with.

Schwartz’s review “Dr. Jekyll and Mr. Hyde: A Short History of Anthrax,” while still discussing virulence factors and mutable pathogenesis, diverges from the articles cited above. It is helpful to quote one particular section in full to get a sense of the way Schwartz uses the novel to narrate the history of the relationship between humans and *B. Anthracis*: “The history of anthrax, as we have seen, is clearly double-faced, reminiscent of *The Strange Case of Dr. Jekyll and Mr. Hyde*, written in 1886 by Robert Louis Stevenson. Like Mr. Hyde, anthrax has brought evil on people. Not only did it kill thousands of animals and human beings since Antiquity, and still does, but it was also turned into a potentially murderous weapon for bacteriological warfare and bioterrorism” (p. 353) [36]. But *B. anthracis* has not exclusively acted as villain to the human race: “Like Dr. Jekyll, it has done a lot of good to humanity, since its study paved the way for the fight against infectious diseases. Indeed, anthrax was the first disease that could be attributed to a specific microorganism, and its study allowed Koch to devise novel staining and cultivation methods, useful for many other bacterial pathogens. In addition, the study of anthrax led to the elaboration of Koch’s postulates that are at the foundations of medical microbiology. The success of the vaccine against anthrax started the science of vaccines in general; the work of Pasteur and his colleagues on anthrax included the formulation of concepts as important as “antibiosis” and species barrier. Moreover, the present day studies on the pathophysiology of the disease, including an investigation of the role of its toxins, have made of *B. anthracis* one of the best models in infectiology” (p. 353) [36]. As with Kendall, there is some irony here in the ascription of a Jekyll and Hyde dualism to *B*. *anthracis*, when it was actually humans (and government programs in the U.S., U.K., and Soviet Union, at least initially) that repurposed the microbe as a bioweapon. That said, Schwartz’s treatment of the microbe as an “actor” or agent in a network, in the Latourian sense [37], of scientists and laboratories that facilitated discoveries not directly related to the microbe itself is in fact a productive frame. We can think of *Jekyll and Hyde* in a similar capacity: how might we reconceive of the novel’s effect on the research that cites it?

## 4. Stevenson’s *Jekyll and Hyde*, the Gothic, and “the Incongruous”

Literature affords a multidimensional quality to the content it describes; that is, it allows for analysis of—and draws attention to—the complexity, ambiguity, and possible overdetermination in its language and context. Literary studies, as a field of research, works to understand the forms, mechanics, history, and impact of literature in all its affordances and limitations. One might say that science, in its efforts to explain the why and how of natural phenomena and enable human technological intervention and manipulations of it, works towards similar ends. But even beyond their possibly coextensive epistemological capacities, fictional representations of science matter to science. They have been documented to influence science in terms of ideation and innovation. They become part of the grammar and vocabulary that shape what is thinkable and knowable, particularly as they are used to articulate theory, experiment, results, and the working objects of science. Turney’s *Frankenstein’s Footsteps: Science, Genetics and Popular Culture*, for example, is one of many studies demonstrating the mutually constitutive relationship between literature and science [38,39,40,41,42,43,44,45,46,47,48,49,50,51,52,53]. Like Mary Shelley’s *Frankenstein; Or, The Modern Prometheus*, *Jekyll and Hyde* has a long history in the explanation and interpretation of various cultural phenomena. Appearing in journalistic, medical, scientific, and criminological prose—giving the story almost the status of a myth—it has become a prêt-à-porter rhetorical and heuristic device with expansive application across multiple domains [41,42,43,54,55,56,57].

Stevenson published *Jekyll and Hyde* in 1886. The novel is composed of three different narratives: one follows Gabriel Utterson, close friend of Dr. Henry Jekyll (who of course doubles as the mysterious Mr. Hyde); Dr. Henry Lanyon, friend of Jekyll, who witnesses and documents the transformation; and Dr. Jekyll, who provides a conclusive “Statement of the Case” in his suicide note, which Utterson finds on the dead body of Mr. Hyde. The scholarship on Stevenson’s novel and its relationship to science (including and apart from the “mad scientist” motif) is vast, delving into its past and current relevance to psychiatry, addiction, neuroscience, evolution, and bioethics, among other areas [40,41,42,43,54,55,56,57]. Although it was published during the emergence of the bacteriological age of Koch and Pasteur, it does not contain any explicit references to microbes. This does not, however, preclude it from contextualization within bacteriology given the era of its publication, its significance to the canon of late Victorian Gothic science fiction, and its idiomatic use as a rhetorical shorthand in scientific publications.

Monsters like Hyde often have the most to tell us about those who name and read them as monsters. Late Victorian Gothic fiction like *Jekyll and Hyde* reflected and critiqued the science, urbanization, industrialization, and imperialism of the late nineteenth century. It employed a number of identifiable conventions: the decayed, labyrinthine corridors of the city; the dangers of playing God through scientific hubris; anxieties of racial degeneration in the face of Darwinian evolution and eugenic science; the medicalization of criminality; and the dissolution of comforting identity categories such as class, gender, sexuality, and even humanity. It also sought to fathom and often challenge the boundary between the normal and the pathological. The Gothic, then, is a fitting genre to explore anxieties related to scientific developments. Its blurring of oppositions resonates with both the challenges of defining virulence, delimiting microbes to “Jekyll’s” or “Hyde’s,” and with the consequences of misusing antibiotic compounds in medicine and agriculture (a misuse present from those compounds’ inception). Two specific dimensions in the novel stand out in terms of the nuanced and imaginative work it could contribute to current antibiotic and microbiological research: the indeterminate descriptions of Hyde and the undefined nature of Jekyll’s compound.

First, with respect to Hyde, nearly every description of the man by those other than Jekyll, while imbued with charged attributes of monstrosity and atavism, struggles to name exactly what about him is so deviant from the norm. Hyde’s degenerate body is a product and reflection of its environment [19,20,21]. The slums of modern London, the “district of some city in a nightmare” (p. 40) [58], shape and enable his unspeakable acts. This is in our very first introduction to the character, when Utterson hears about how this monstrous figure tramples a young child in Soho near Jekyll’s residence: “He is not easy to describe. There is something wrong with his appearance; something displeasing, something down-right detestable. I never saw a man I so disliked, and yet I scarce know why. He must be deformed somewhere; he gives a strong feeling of deformity, although I couldn’t specify the point. He’s an extraordinary looking man, and yet I really can name nothing out of the way. No, sir; I can make no hand of it; I can’t describe him. And it’s not want of memory; for I declare I can see him this moment” (p. 12) [58]. Here we see a general sense of abjection and uncanniness around Hyde, but few specifics. This is not for lack of observation or recollection: Hyde’s indefinable monstrosity exceeds the language to describe it. Hyde’s elusive form is consonant with the challenges of defining virulence as an attribute of pathogens: as Casadevall contends, “the question ‘what is a pathogen?’ is rooted in pathogen-centered views of microbial pathogenesis. This question cannot be answered without also defining a host because microbial virulence is not independent of host susceptibility. Hence, the question, ‘what is a pathogen?’ and its counterpart, ‘what is a host?’ are distractions from the more relevant and answerable question: “what is the outcome of the host-microbe interaction?” (p. 4) [59]. In the same way we consider host-microbe interactions as a mode of relationality and a process, we can also consider Jekyll (along with the other protagonists) and Hyde (the central antagonist) as an enmeshed, indivisible assemblage rather than a juxtaposition of polarized, discrete entities. We cannot really understand how Hyde looks or operates without accounting for Jekyll, and late Victorian London. Taking one further metacognitive step back, we might likewise account for the use of the novel *Jekyll and Hyde* in relation to that which it is being used to represent: the dynamic interrelation between humans and contextually pathogenic microbes. The novel serves as a heuristic instrument in the medium of the scientific journal, but like the Gothic genre itself, the novel’s use carries an excess of meaning that refuses to be delimited, or reductively disassembled into parts. I would argue, however, that this is not a limiting factor of the use of fiction in scientific prose but rather an affordance—it allows for and invites more expansive thinking.

Second, it is important to note that Jekyll’s drug is not the agent of his demise. It is not essentially good or bad, not medicine or poison. As Jekyll admits, “The drug had no discriminating action; it was neither diabolical nor divine; it but shook the doors of the prison house of my disposition; and like the captives of Philippi, that which stood within ran forth. At that time my virtue slumbered; my evil, kept awake by ambition, was alert and swift to seize the occasion; and the thing that was projected was Edward Hyde. Hence, although I had now two characters as well as two appearances, one was wholly evil, and the other was still the old Henry Jekyll, that incongruous compound of whose reformation and improvement I had already learned to despair” (p. 116) [58]. Its dual effects, first therapeutic then toxic, echo a foundational principle of toxicology: “the dose makes the poison” [60]. Indeed, how compounds get categorized, especially in the early development of pharmacology and toxicology in the nineteenth century, is often a question of how much is used and to what end. While the pharmacological ambivalence in the novel is often read in terms of addiction, the mechanism of downregulation associated with addiction may actually be counterproductive in the microbiological register, as it mirrors a common misunderstanding of the nature of antibiotic resistance—that the person becomes resistant to the drug. More useful is being attentive to the degree (how much) and capacity (to what end) in which antibiotics are used. Take for example the overuse or subtherapeutic misuse of antibiotics as growth-promoting agents in livestock. In this example the drug is not the problem; human misuse is.

Considered through a humanities lens, the dynamic between Jekyll, Hyde, the “incongruous compound,” and their collective milieu can be seen to capture the human impact on bacterial life as well as the impact of bacterial life on the human, without ever losing sight of broader ecological contexts. The novel can be read as the underscoring of the same epistemic challenge forced by antibiotic resistance. The writing of human history into bacterial life, in the words of Landecker, has occasioned “a decentering of the very units of analysis that we might use to decide what is human, non-human, animal, viral, species, bacterial, embodied, environmental, intentional, or engineered in the first place” (p. 5) [61]. In the face of this resistance—and perhaps through a more careful engagement with language and figurations we use to apprehend and describe it—we must continually acknowledge the way we are inextricably, dynamically bound to microbial life, and, moreover, how our attempts to technocratically expunge microbes has come back to haunt us. It is incumbent upon us to appreciate not just the technology but the thinking that got us here.

## 5. Conclusions

Recently there has been an impulse to include humanistic and social scientific dimensions in scientific and biomedical research, yet this is often reduced to the form of a checkbox on a grant application. Certainly, readers of a journal like *Antibiotics* realize the utility in—the necessity of—working with different disciplines, especially in light of a severe and multifarious problem like antibiotic resistance. As we develop the field of anti-virulence in response to antibiotic resistance, an appreciation of the nuances of rhetorical devices such as allusion and figuration, as well as narrative modes and their historical context, is crucial. Acknowledging that the very logic of antibiotics, or *antibiosis* (“against life”), is imbued with anthropocentric metaphors of militarism, which have facilitated the overuse of antibiotics [62,63,64,65], and has stemmed at least in part from literary texts, we must grant the premise of fields like science and technology studies that hold there is no hermetic boundary between science and culture [44,66,67]. The common thread here is language. Like any technology, language—which is used in science both to discover and explain its phenomena—constrains thinking as much as it enables it. As Locke suggests in *Science as Writing*, “Science is the language it utters itself in. Surely now science must consider what it is saying, how it is saying it, and why it is saying it the way that it is” (p. 206) [68]. Attending to these dimensions of science can help researchers be more attuned to the impact their writing has on their research; this attunement should inflect how scientists talk, think, and communicate about their work to each other.

There have already been numerous instances and calls to consider antibiotic resistance across disciplines, and not just scientific ones at that [69,70,71,72,73,74,75]. Following these prompts, along with the invitation by Brannon and Mulvey to dig deeper into the heterogeneity of microbes, I would ask readers of this special issue to “dig deeper” into the past and possible future uses of literature, language, and history in their writing on antibiotics and microbiology. A good faith interdisciplinary effort would entail going to literature not only for the vivifying and stylistic reinforcement of an existing argument, but also to access potential re-imaginings of how scientific research is thought of and framed.

For example, engaging with Stevenson’s novel beyond its now-mythic shorthand for duality provides specific advantages for the interdisciplinary study of pathogens and antibiotics. It offers ethical and conceptual tools for the scientific work seeking to understand the nature of microbes, for the engineering work seeking to develop new alternatives to antibiotics, and for the clinicians who deploy those technologies. From an ethical perspective, it is not just a reminder of Frankensteinian (Faustian) hubris; *Jekyll and Hyde* also expresses the irreversibility of certain technoscientific interventions, which raises vital questions in terms of ecological, distributive, and intergenerational justice. Certainly, the technoscientific intervention of industrialized antibiotics has forever changed our social determinants of health, our ecology, and our future, not unlike climate change: “‘We used to think a certain way about antibiosis and pathogens. And then we changed the future.’ What we thought we knew became the biology under study: the solution has become the problem. Not all sciences confront the contours of their past logics as mass irruptions at global scale of thoroughgoing changes in forms of life. Bacterial life today is appearing as a specific instantiation of the biology of the Anthropocene: human efforts to control life’s productivity become the matter of the world” (p. 5) [61]. Apropos of the novel’s Gothic mode, in our moment, while we might seem to be through with the past by way of innovation and a transformed epistemic paradigm, our past over- and misuse of antibiotics, now inscribed in the bacterial resistome [61,76], is clearly not through with us.

What are the technological and cultural changes we must marshal in order to stem the tide of antibiotic resistance? Literature, through its multiplicity of perspectives, narrative engagements, and dialogical operations, fosters the interpretive flexibility and expandability that lends itself to such questions, but only when it is treated as an object of inquiry that requires training, methodology, and at least a preliminary review of extant scholarship—just as with *C. Diphtheria*, *pfra*, β-lactamases, or any other object of study relevant to antibiotic research. Thinking about science with an interdisciplinary sensitivity to literary, historical, and cultural study can expand and perforate the tunnel vision that necessarily comes with a focused and defined research question.
